# Can sports cartoon watching in childhood promote adult physical activity and mental health? A pathway analysis in Chinese adults

**DOI:** 10.1016/j.heliyon.2022.e09417

**Published:** 2022-05-14

**Authors:** Xing Zhang, Matthew H.E.M. Browning, Yong Luo, Hansen Li

**Affiliations:** a Institute of Sports Science, College of Physical Education, Southwest University, Chongqing 400715, China; b Department of Basketball and Volleyball, Chengdu Sport University, Sichuan 610041, China; c Department of Park, Recreation, and Tourism Management, Clemson University, Clemson, SC 29634, USA

**Keywords:** Sports cartoon, Child, Childhood, Mental health, Physical activity, Sports

## Abstract

Physical activity is essential to maintain physical and mental health. Unfortunately, insufficient physical activity has become a common phenomenon worldwide in the past decade, and the absence of interest in physical activity is assumed a reason. Therefore, strategies for fostering interests and promoting public engagement are in need. We conducted a questionnaire survey during September 2021 that involved 1202 adults to capture data on sports and relevant cartoon watching experiences during childhood; Intensity and frequency of physical activity, depression (PHQ-9), and anxiety (GAD-7) during adulthood. Structural equation modeling was employed to examine pathways between watching sports cartoon in childhood and physical activity and mental health in adulthood. The results suggest that childhood sports cartoon watching may have indirect and positive impacts on adult physical activity and, in turn, on mental health. Childhood sports experience is a critical mediator role linking childhood sports cartoon watching, adult physical activity, and adult mental health. In conclusion, watching sports cartoon may help cultivate sports interests and promote children's participation in sports, and the sports experiences may be a continuous and beneficial factor that connects childhood and adulthood. These findings indicate a framework to understand the durative positive impact of cartoons and maybe other children's film and television works.

## 1 Introduction

Depression and anxiety are major mental health problems that threaten life quality and even lead to severe consequences, such as self-mutilation and suicide (Hansson, 2002; Resch et al., 2008; Ross and Heath, 2002). Nowadays, more than a billion people worldwide are suffering from mental health problems (Kochhar et al., 2020; Rehm and Shield, 2019). Many studies suggest that chronic stress in urban living is an important reason for the mental health issue, which may result from common negative experiences (Uliaszek et al., 2012), such as diseases (Huang et al., 2010), academic performance (Alvi et al., 2010; Saravanan and Wilks, 2014), and overload work (Alvi et al., 2010; Sreeramareddy et al., 2007).

Physical activity (PA), the most popular health-boosting strategy, shows great potential in reducing stress and mental health problems (Chekroud et al., 2018; Ohta et al., 2007). However, a population-based study involving 1.9 million participants underlines that the general public's physical activity level has been declining over the past few years (Guthold et al., 2018). There is an urgent need to encourage the general public to engage in sports and any kind of physical activity (Guthold et al., 2018). To achieve this goal, fostering sports interests can be a realistic method (Moschny et al., 2011).

Childhood is a critical period for culturing sports interests. Positive sports participation in childhood may help maintain adults’ interests in sport and promote physical activity (Miller and Siegel, 2017; Taylor et al., 1999), and these benefits may be durative. For example, studies suggest that sports experiences and skills acquired in childhood may contribute to sports participation adaptions for new modes of physical activity during adulthood (Tammelin et al., 2003). Therefore, starting sports in childhood can be significant.

Cartoons are highly attractive for children and usually take most of their leisure time (Hassan and Daniyal, 2013). Currently, the positive effect of cartoons on children is gradually receiving more attention. Identified benefits include reducing pain (Düzkaya et al., 2021), easing fear (Düzkaya et al., 2021), and regulating food preference (Hémar-Nicolas et al., 2021). Sports cartoons are found to cultivate children's sports interests and motivate participation (Yu and Xu, 2011). The developed sports interests may be lasting and even influence physical activity participation and mental health during adulthood (Gerber and Pühse, 2009; Harris et al., 2006). Therefore, exploring the impact of sports cartoons on children's sports participation and later life course may advance the understanding the role of cartoon education and the connections between childhood and adulthood.

## 2 Conceptual framework

**Figure 1:**
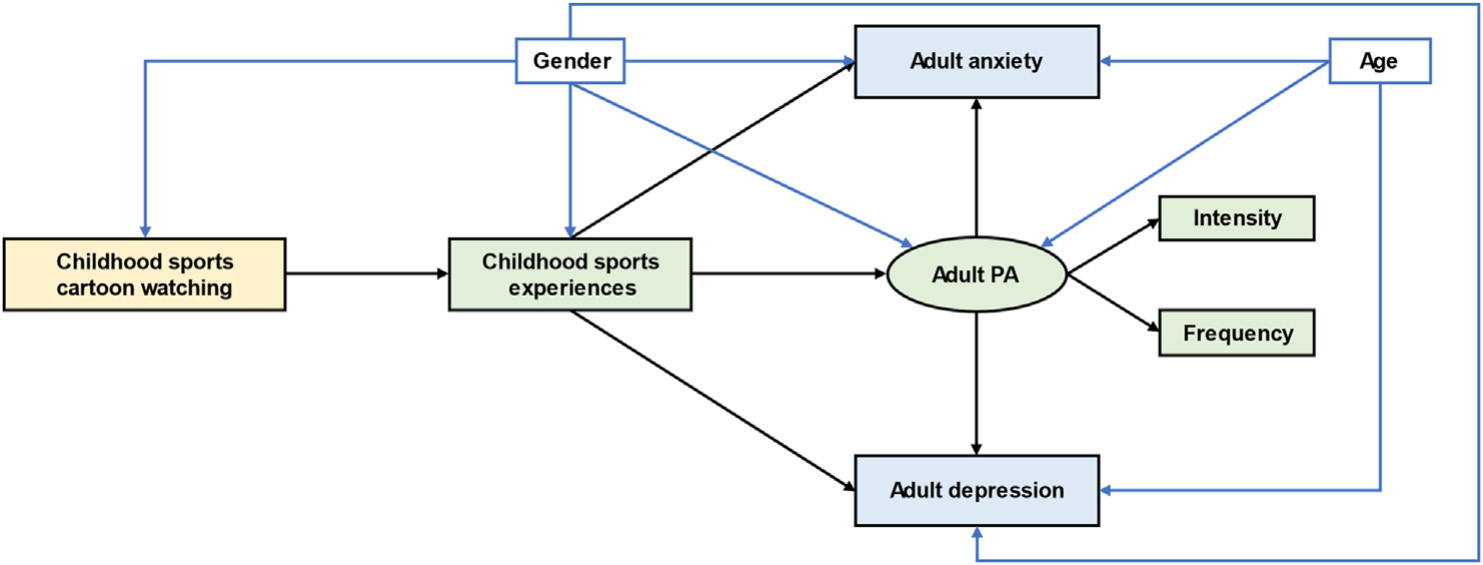
Conceptual model of childhood sports cartoons watching – adult mental health pathways.

Cartoons can impact children's behaviors and habits (Hassan and Daniyal, 2013). Sports cartoons may attract adolescents and children to actively participate in sports (Yu and Xu, 2011), thus increasing their sports experiences. According to Merkel (2013), sports experiences during childhood can help children develop strategies to cope with stress from adverse experiences, indicating a life-long mental benefit. Besides, sports habits cultivated in childhood can be a lasting beneficial factor for mental health. Sports experiences during childhood may motivate adults to adhere to physical activity (Batista et al., 2019). Since physical activity plays an essential role in reducing depression and anxiety (Gerber and Pühse, 2009; Harris et al., 2006), childhood sports experiences may benefit adult mental health by motivating adults to participate in physical activity. Based on the above clues, we established our conceptual model in which childhood cartoons impact adult anxiety and depression through fueling childhood sports experiences and adult physical activity (Figure 1). Control variables including gender and age were included in the framework because they may alter the benefits of physical activity (Van Tuyckom et al., 2010). Confounding pathways were established according to corresponding time sequences. Specifically, only gender was linked to all core variables (childhood sports cartoon watching, childhood sports experiences, adult PA, adult anxiety, and adult depression) because it may impact individuals through the life course. We hypothesized as the following:

(1) Childhood sports cartoon watching may impact adult PA through childhood sprots experience;
(2) Childhood sports cartoon watching may impact adult mental health through childhood sprots experience and adult PA.

## 3 Materials and methods

### 3.1 Procedure and participants

We conducted an online questionnaire survey during September 2021. We used a snowball strategy to spread recruiting information. We invited 50 college students from Sichuan Agriculture University, Chengdu Sport University, and Southwest University to spread the message through their social circles and any other possible online chat groups. The research topic was introduced as “Investigating childhood behaviors and current health,” and our research questions were not disclosed.

A consent was demonstrated at the cover of the questionnaire to inform participants of their rights in this research and how their data would be used. All participants were required to use a WeChat account (the most popular social network in China) bonded with their legal identities to participate. We employed a human verification technology to ensure that each of the questionnaires was carefully completed by a person.

To maximize the response rate, we offered a 5 RMB (approximately 0.8 USD) award for participation. Eventually, a total of 1202 returned and qualified questionnaires were included for the analysis. The study was approved and supervised by the Ethics Review Board of Southwest University, China.

### 3.2 Measurements

#### 3.2.1 Depression

Depression was measured using a Chinese version of the Patient Health Questionnaire (PHQ-9) (Wang et al., 2014b). The questionnaire's reliability and validity have been confirmed in the general population (Wang et al., 2014b; Yu et al., 2012). The PHQ-9 consists of nine questions based on the nine DSM-IV criteria for a major depressive episode. Each of the questions requires patients to select the frequency of the depressive symptoms they experienced in the two weeks before the investigation. Items were rated on a 4-point Likert scale (0 = not at all and 3 = nearly every day).

#### 3.2.2 Anxiety

Anxiety was measured using a validated Chinese version of Generalized Anxiety Disorder (GAD-7) (Garabiles et al., 2020). The scale is a 7 item self-rating instrument. Each item described one of the typical symptoms of GAD and was evaluated by the frequency in which that symptom emerged over the last two weeks. Items were rated on a 4-point Likert scale (0 = not at all and 3 = nearly every day).

#### 3.2.3 Physical activity

We followed the method of Brailovskaia and Milton et al. (Brailovskaia et al., 2018; Milton et al., 2011) and investigated the level of physical activity through frequency and intensity. Questions were worded as follow:

Frequency: “How often did you perform physical activity last month?” Answers were captured using a 7-point Likert (1 = never and 7 = almost every day).

Intensity: "Describe the general intensity of your physical activity in last month?” Answers were captured using a 7-point Likert (1 = very easy, 7 = exhausted).

#### 3.2.4 Childhood sports experiences

The following questions measured childhood sports experiences:

“During your childhood (≤12 years), how often did you participate in sports?” A 7-point Likert scale was used to collect answers, where 0 = never, 6 = almost every day.

#### 3.2.5 Childhood sports cartoons watching

The following questions measured childhood sports cartoon watching:

“During your childhood (≤12 years), how often did you watch sports cartoons?” A 7-point Likert scale was used to collect answers, where 0 = never, 6 = almost every day.

### 3.3 Statistical analysis

Internal reliabilities of the measuring questionnaires were analyzed with Cronbach's alpha. Cronbach's alpha that greater than 0.70 was considered acceptable (Wang et al., 2014a).

Structural equation modeling (SEM) was employed to examine the pathways of the conceptual model (Anderson and Gerbing, 1988), and the maximum-likelihood method was applied for the asymptotically unbiased, consistent, and efficient estimators (Walumbwa et al., 2005). The direct and indirect effects among the conceptual model were analyzed via the bootstrap method (5000 times sampling and 95% confidence interval) (Hayes, 2009). The goodness of fitting was assessed by the following items: value of χ2/df < 3.00, Goodness-of-fit index (GFI) > 0.90, Comparative fix index (CFI) > 0.90, Normed-fit index (NFI) > 0.90, Tucker-Lewis index (TLI) > 0.90, Adjusted Goodness of Fit Index (AGFI) > 0.90, Bollen's incremental fit index (IFI) > 0.90, and Root mean square error of approximation (RMSEA) < 0.08. The factor loading for latent variables of the conceptual model over 0.45 was considered acceptable (Hair et al., 2009. Print). An indirect effect (i.e., a product of coefficients for the constituent links) that significantly exceeded zero was evidence of mediation (Hayes, 2017; Zhao et al., 2010).

We tested the initial model and observed acceptable model fitting and factor loading values (Supplementary Table S1). After that, we removed non-significant pathways according to their t-values (t < 1.96) to adjust our initial model. In total, we tested eight models that were consistent with our conceptual framework. We used the Akaike information criterion (AIC) to compare the fit of competing or alternative models (Moss, 2009). Consequently, one of the eight models was selected as the final model according to the acceptable model fit and lowest AIC.

Statistical analysis was performed using SPSS 25.0 and AMOS 23 (SPSS Inc. IL, Chicago, USA). A two-sided *p* < 0.05 was considered statistically significant except as noted above.

## 4 Results

### 4.1 Validation of measurements

The Cronbach's alpha for Stress (PSS-10), Depression (PHQ-9), Anxiety (GAD-7) was 0.76, 0.92, and 0.94, respectively, indicating ideal internal consistency.

### 4.2 Characteristics of the study population

A total of 799 (66.47%) males and 403 (45.45%) females were involved in the current study, and all the participants are adults (age >18). Only 7.57% of the participants reported no cartoon watching during childhood (Table 1). Also, only 3.16% of the participants reported no sports experience during childhood.

**Table 1:** Summary statistics of the study population.

Variables	Category	Mean (SD)	Percentage
Gender	Male		799 (66.47%)
	Female		403 (43.53%)
Age (years)		27.48 (7.11)	
Childhood sports cartoons experiences	Never		91 (7.57%)
	Seldom		192 (15.97%)
	Occasionally		310 (25.79%)
	Sometimes		338 (28.12%)
	Usually		123 (10.23%)
	Almost every day		148 (12.32%)
Childhood sports experiences	None		38 (3.16%)
	Seldom		208 (17.30%)
	Occasionally		352 (29.28%)
	Sometimes		414 (34.44%)
	Usually		77 (6.40%)
	Almost every day		113 (9.40%)
Adult physical activity	Frequency (score)	3.45 (1.85)	
	Intensity (score)	2.55 (1.41)	
Adult depression (PHQ-9 score)		4.99 (5.08)	
Adult anxiety (GAD-7 score)		4.27 (4.63)	

### 4.3 SEM model

**Figure 2:**
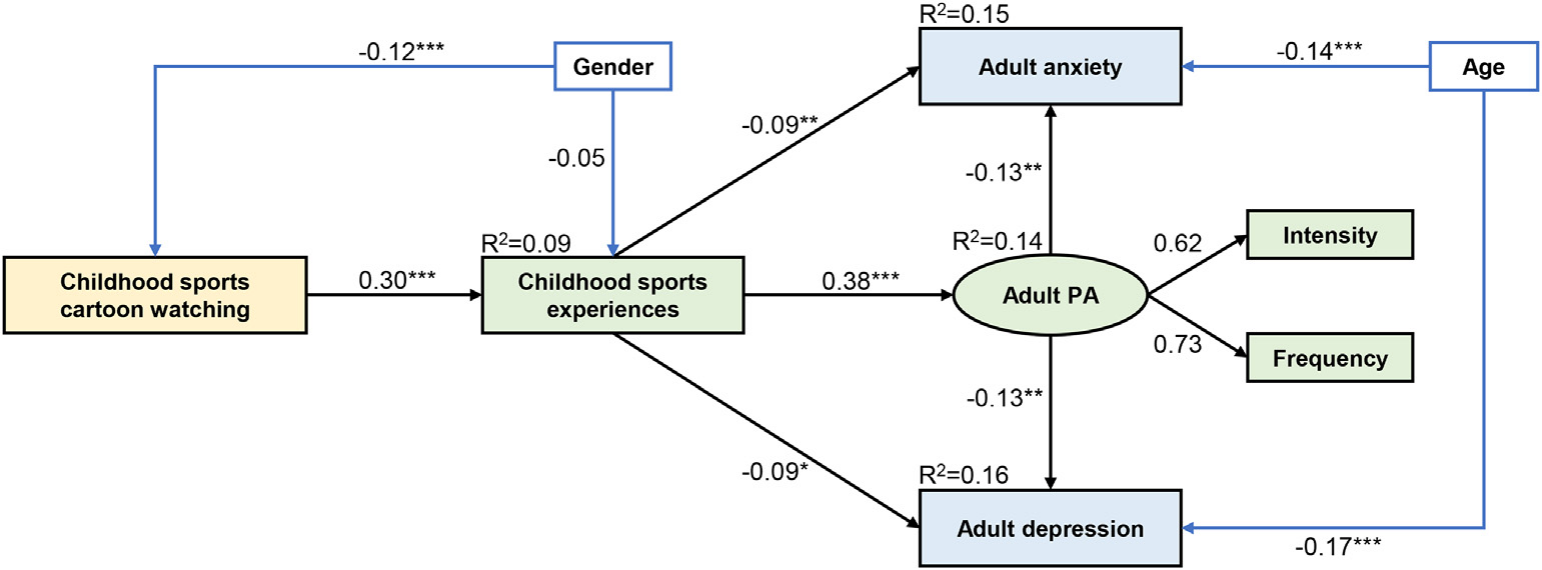
The final SEM model with standardized effects (β). ∗, *p* < 0.05; ∗∗, *p* < 0.01; ∗∗∗, *p* < 0.001. R^2^, explained variance. PA, physical activity

We selected the model with best fits as the final model (χ2/df = 2.239, p = 0.005, GFI = 0.994, AGFI = 0.983, NFI = 0.981, IFI = 0.989, TLI = 0.978, CFI = 0.989, and RMSEA = 0.026). Besides, the factor loadings were ranged from 0.62 to 0.73, which were acceptable (Figure 2).

Table 2 and Figure 2 demonstrate the direct and indirect pathways in the final model. No significant direct pathway was found from childhood sports cartoons to adults' physical activity, depression or anxiety. However, several distinctive indirect pathways were identified from the final model. First, we found a positive indirect effect of childhood sports cartoons on adult physical activity via promoting childhood sports experiences (β = 0.114; *p* < 0.001). Second, we found indirect pathways between childhood sports cartoons and adult depression (β = -0.026; *p* = 0.011) and anxiety (β = -0.029; *p* = 0.003) via childhood sports experiences. Moreover, a longer route comprising the above pathways was also identified, childhood sports cartoons positive effect on adults' depression (β = -0.015; *p* = 0.002) and adults' anxiety (β = -0.015; *p* = 0.002) via childhood sports experience and adults' physical activity. Regarding participants’ characteristics, we found that gender had a significant effect on childhood sports cartoons experiences (β = -0.117, *p* < 0.001): males experienced more sports cartoons than females during childhood. Besides, age had a significant and negative effect on adult depression (β = -0.166, *p* < 0.001) and anxiety (β = -0.139, *p* < 0.001).

**Table 2:** Standardized direct and indirect pathways of the model.

Effect	Pathways	β (95% CI)	*p*-value
	C→PA	0.114 (0.084, 0.148)	<0.001
Total	C→D	-0.041 (-0.064, -0.022)	<0.001
	C→A	-0.044 (-0.066, -0.025)	<0.001
	C→S	0.302 (0.246, 0.363)	<0.001
	S→PA	0.377 (0.300, 0.450)	<0.001
Direct	S→D	-0.087 (-0.157, -0.018)	0.014
	S→A	-0.095 (-0.157, -0031)	0.004
	PA→D	-0.130 (-0.212, -0.045)	0.002
	PA→A	-0.134 (-0.219, -0.049)	0.002
	C→S→PA	0.114 (0.084, 0.148)	<0.001
	C→S→PA→D	-0.015 (-0.027, -0.005)	0.002
Indirect	C→S→PA→A	-0.015 (-0.028, -0.006)	0.002
	C→S→D	-0.026 (-0.050, -0.006)	0.011
	C→S→A	-0.029 (-0.051, -0.010)	0.003

Note: C, childhood cartoon watching; S, sports experiences; PA, physical activity; A, anxiety; D, depression.

## 5 Discussion

### 5.1 Role of childhood cartoon watching on adult physical activity

We did not find evidence of the direct impact of childhood cartoon watching on adult physical activity participation. Instead, we found an indirect association between them through childhood sports experience, and the mediation effect was significant (β = 0.114; *p* < 0.001). These results are in line with our theoretical framework, partially explaining the positive associations previously identified between childhood cartoons and adult physical activity (Perkins et al., 2004; Taylor et al., 1999). Our results suggest that the direct impact of cartoon watching in childhood may not be durative. This may be because individuals’ attitudes towards cartoons can completely change over their lifespan (Fernandes and Oswald, 2005). Nevertheless, the meditation effect suggests that childhood cartoon watching may lead to higher levels of childhood sports experiences and, in turn, to higher levels of physical activity as an adult. This result implies that sports cartoons may motivate children to engage in sports and therefore develop a sports habit, which may provide a mechanism to understand a previous study, where participation in sports during childhood was positively associated with physical activity during adulthood (Batista et al., 2019).

Though few studies focused on the life-long impacts of sports cartoon watching, many other studies have identified connections between childhood and adulthood. For example, childhood residential environments, adverse experiences, and intimate relationships can impact lives in midlife or even further (Astrup et al., 2017; Flouri, 2004; Springer, 2009). Bures (2003) argued that childhood might impact life later through some health-related behaviors that are functional during the whole life. Based on our findings, sports habits cultivated in childhood sports experiences can be such a health-related behavior. Since childhood sports cartoons may be a source of children's participation in sports, using cartoons to motivate children to adhere to physical activity may be a feasible strategy. Nowadays, children are spending more time watching TV, which has threatened their physical and mental health (Düzkaya et al., 2021; Hémar-Nicolas et al., 2021). However, though many methods have been proposed to shorten the time in front of the TV, insufficient efforts have been made to find health-promotion TV content for children (Salmon et al., 2007). As described in the Introduction, cartoons are highly attractive for children, and children usually imitate the characters of the cartoons. Many studies have documented the risks of cartoon imitation, such as violence and risky behaviors (Hassan and Daniyal, 2013; Paluska and Schwenk, 2000). However, the cartoon imitation can be positive if the content is healthy and helpful. Sports cartoons may help children learn about sports and offer them a positive character to imitate. In the past 20 years, many sports cartoons (animation) that originated from Japan have affected children and teenagers worldwide, such as Slum Dunk (basketball), Captain Tsubasa (football), and Touch (baseball). Although no statistical data, many professional athletes have reported being motivated by those cartoons during childhood. For example, Sergio Agüero, a national football player of Argentina, stated that he has been encouraged by cartoon Captain Tsubasa and became a professional player, which may underline the bright side of cartoon imitation. In China, cartoon is regarded as a method to educate children (ZhongPing and Yan, 2010). All the cartoons on TV or the internet are released under the supervision of the National Radio and Television Administration of China. Due to Chinese policies on cartoon production and education, many people have been dedicated to producing proper cartoons to help children build a sense of justice and foster health-promotion behaviors (China, 2008). Based on our findings, policymakers of China and maybe other regions are recommended to use sports-based cartoons or other film and television works to help foster sports habits in children.

### 5.2 Impact of childhood cartoon watching on adult mental health

In the current study, we did not find a direct impact of childhood cartoon watching on adult anxiety and depression. In contrast, we found childhood cartoon watching impacted the two mental health issues through multiple mediators. Specifically, childhood sports experience was a critical mediator, which directly impacted the two mental health issues, also indirectly impacted them through adult physical activity. The direct paths between childhood sports experience and mental health issues are in line with our conceptual framework. Empirically, physical activity may help to develop mental resilience to cope with stress and adverse experiences (Gerber and Pühse, 2009; Harris et al., 2006), which is reported to possibly cause life-long impacts (Lee et al., 2019). Accordingly, this path may explain how childhood sports can reduce mental health issues without the need for current participation in physical activity. On the other hand, the longer paths mediated by adult physical activity are also in line with our hypothesized pathway, underling the benefits of physical activity on mental health.

Generally, our findings suggest that childhood cartoon watching may promote adult physical activity engagement and benefit mental health through indirect impacts. Cartoons are popular and a part of children's lives, which can be considered as a strategy for public health promotion.

## 6 Limitation

We used self-reported data. Potential reporting bias cannot be ruled out. Some people may have been afraid to disclose their poor mental health conditions, so the depression and anxiety levels could have been underestimated.

We used single items to investigate childhood sports and cartoon experiences because there is no appropriate instrument or pre-established dimensions for these variables. Future studies may need to address this issue by developing new questionnaires.

We recruited participants online. It is possible that participants who tended to use screens, including TVs in childhood and smartphones or PCs in adulthood, have participated in this investigation. Therefore, our data may not have represented the general public. Future research may need to use offline methods to recruit participants.

We also could not obtain real-time data for childhood variables, and the conditions in childhood were simply recalled by participants, which may lead to strong bias given the interval between the investigated periods of childhood and adulthood. In addition, our model was examined using data captured in a cross-sectional investigation. The causal relationships between variables were based on theoretical assumptions drawn from previous literature. This is an inherent limitation of SEMs using cross-sectional data. Thus, longitudinal studies are warranted to re-examine the identified pathways. For example, tracking habits of cartoon watching and sports participation in pupils and testing their PA levels in the future can be a practical method.

## 7 Conclusions

The current study presented a mechanism to explain how sports cartoons experiences during childhood may benefit adult physical activity and mental health. Our findings suggest that sports cartoons experiences during childhood may indirectly promote physical activity engagement and mental health during adulthood. Although we did not investigate children, our retrospective results indicate that sports cartoons play beneficial roles in motivating children to participate in sports, which may further help develop sports habits to exert life-long benefits. As cartoon is an essential part of children's lives, policymakers are recommended to use better sports cartoons as a strategy to promote public physical activity participation.

## Declarations

### Author contribution statement

Xing Zhang: Conceived and designed the experiments; Performed the experiments; Analyzed and interpreted the data; Contributed reagents, materials, analysis tools or data; Wrote the paper.

Matthew H.E.M. Browning; Yong Luo: Analyzed and interpreted the data; Wrote the paper.

Hansen Li: Conceived and designed the experiments; Analyzed and interpreted the data; Wrote the paper.

### Funding statement

This research did not receive any specific grant from funding agencies in the public, commercial, or not-for-profit sectors.

### Data availability statement

Data will be made available on request.

### Declaration of interests statement

The authors declare no conflict of interest.

### Additional information

No additional information is available for this paper.
