# Supplementary material for: Can sports cartoon watching in childhood promote adult physical activity and mental health? A pathway analysis in Chinese adults
Source: Heliyon. 2022 May 14;8(5):e09417. doi: 10.1016/j.heliyon.2022.e09417 (PMC9123224; doi:10.1016/j.heliyon.2022.e09417)
Supplement: Supplementary [file mmc1.docx]

| **Model** | **χ2** | **df** | ***p*** | **χ2/df** | **AIC** | **BIC** |
| --- | --- | --- | --- | --- | --- | --- |
| 0 (Initial) | 20.846 | 7 | 0.004 | 2.978 | 78.846 | 226.506 |
| 1 | 21.362 | 8 | 0.006 | 2.670 | 77.362 | 219.931 |
| 2 | 22.852 | 9 | 0.007 | 2.539 | 76.852 | 214.329 |
| 3 | 24.532 | 10 | 0.006 | 2.453 | 76.532 | 208.917 |
| 4 | 26.141 | 11 | 0.006 | 2.376 | 76.141 | 203.434 |
| 5 | 27.748 | 12 | 0.006 | 2.312 | 75.748 | 197.950 |
| 6 | 29.623 | 13 | 0.005 | 2.279 | 75.623 | 192.733 |
| 7 (Final) | 31.341 | 14 | 0.005 | 2.239 | 75.341 | 187.359 |
| 8 | 32.438 | 20 | 0.039 | 1.622 | 82.438 | 209.732 |

**Table S1.** Fitting indices of modified models


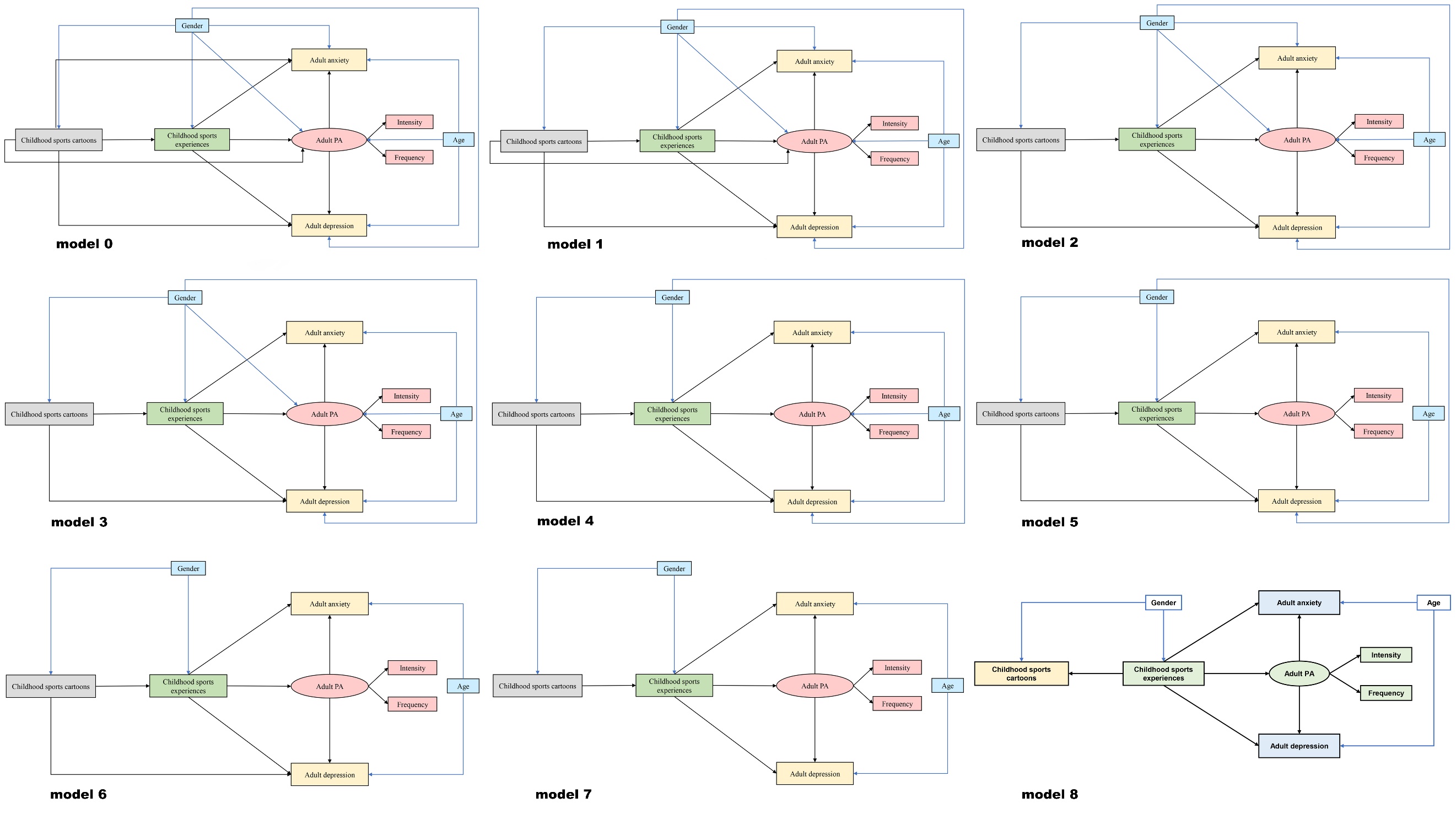


**Figure S1.** Modified model 0 to model 8
